# Meta-omics characteristics of intestinal microbiota associated to HBeAg seroconversion induced by oral antiviral therapy

**DOI:** 10.1038/s41598-021-82939-1

**Published:** 2021-02-05

**Authors:** Yu-Li Zeng, Lei Qin, Wen-Jun Wei, Hong Cai, Xiao-Fang Yu, Wei Zhang, Xiao-Lu Wu, Xiao-Bin Liu, Wei-Ming Chen, Pan You, Mei-Zhu Hong, Yaming Liu, Xuan Dong, Ben-Chang Shia, Jian-Jun Niu, Jin-Shui Pan

**Affiliations:** 1 Department of Gastroenterology, Zhongshan Hospital Xiamen University, Xiamen, Fujian China; 2 School of Statistics, University of International Business and Economics, Chaoyang District, Beijing, China; 3 Department of Hepatology, Xiamen Hospital of Traditional Chinese Medicine, Xiamen, Fujian China; 4 Department of Research Institute, The Fifth Hospital of Shijiazhuang, Shijiazhuang, Hebei China; 5 Department of Infectious Diseases, The First Affiliated Hospital of Xiamen University, Xiamen, Fujian China; 6 Center of Clinical Laboratory, Zhongshan Hospital Xiamen University, Xiamen, Fujian China; 7 Department of Traditional Chinese Medicine, Zhongshan Hospital Xiamen University, Xiamen, Fujian China; 8 Graduate Institute of Business Administration, College of Management, Fu Jen Catholic University, New Taipei City, 24205 Taiwan; 9 Liver Research Center, The First Affiliated Hospital of Fujian Medical University, Fuzhou, Fujian China

**Keywords:** Microbiology, Medical research, Gastroenterology, Hepatology

## Abstract

Tenofovir and entecavir are currently designated as the preferred oral antiviral drugs for chronic hepatitis B. However, only less than 40% of patients can achieve HBeAg seroconversion. We aim at investigating the role of intestinal microbiome in HBeAg seroconversion induced by oral antiviral therapy and describe multi-omics characteristics of HBeAg seroconversion associated intestinal flora. In this study, we prospectively collected fecal samples at baseline from the patients with HBeAg positive chronic hepatitis B who would have oral antiviral therapy. 16S rDNA sequencing and metabolomics were performed. We identified HBeAg seroconversion-related microbial signature and constructed prediction model for HBeAg seroconversion. Thirty-seven of these subjects achieved HBeAg seroconversion within 156 weeks after the initiation of oral antiviral therapy, while 41 subjects remained HBeAg positive even after over 156 weeks of therapy. A computational statistical and machine learning approach allowed us to identify a microbial signature for HBeAg seroconversion. Using random forest method, we further constructed a classifier based on the microbial signature, with area under curve being 0.749 for the test set. Patients who achieved HBeAg seroconversion tended to have lower abundance of certain fecal metabolites such as essential amino acids, and several dipeptides. By analyzing the fecal microbiota from the patients with and without HBeAg seroconversion, we showed intestinal microbiome play a critical role in HBeAg seroconversion induced by oral antiviral therapy. We also identified intestinal microbial signature that is associated with HBeAg seroconversion after oral antiviral therapy.

## Introduction

For hepatitis B virus (HBV) e-antigen (HBeAg)-positive CHB patients, HBeAg seroconversion is a prerequisite for a definite course of oral antiviral treatment^1,2^. Unfortunately, the rate of HBeAg seroconversion following 1 year of treatment is less than ideal for currently available antivirals. The seroconversion rates for pegylated interferon and entecavir are 29–36%^3,4^, and 21%^5^, respectively. After 5 years of therapy, the ratio of HBeAg seroconversion related to entecavir slightly increases to 23%^6^. Similarly, HBeAg seroconversion occurs in 21% of patients following 48 weeks of Tenofovir disoproxil fumarate (TDF) treatment^7^, and the rate increases to 29.9% after 5 years of treatment^8^. Thus, for both entecavir and TDF, HBeAg clearance or seroconversion only occurs in a minority of patients even after multiple years of antiviral therapy. Furthermore, there are few or no additional benefits for extending the antiviral therapy if the patients fail to achieve HBeAg clearance or seroconversion within the first year. Thus, it is critical to screen out patients who may achieve HBeAg seroconversion before the initiation of oral antiviral therapy and provide alternative treatment such as interferon-based therapy for those who show fewer chances for HBeAg seroconversion.

Recently, Chou et al.^9^ found that the intestinal microbiota plays a critical role in the age-related immune clearance of HBV. Consistent with this, the study by Ren et al.^10^ indicated that manipulation of gut microbiota might accelerate HBeAg clearance in the patients who remain HBeAg-positive after years of antiviral therapy. The “leakage hypothesis” proposed by Tremaroli et al.^11^ linked the presence of microbial products in the bloodstream with the onset and progression of liver diseases. Even though intestinal microbiota seems to play a pivotal role in the clinical outcome of CHB, no uniform pattern of gut microbiota related to CHB has been confirmed. In this study, we therefore aimed to determine the link between intestinal microbiota and HBeAg seroconversion in a well-characterized cohort of CHB patients.

## Materials and methods

### Study approval

The Medical Ethics Committee of the Medical College of Xiamen University approved this survey. All participants provided written informed consents. Patients or the public WERE NOT involved in the design, or conduct, or reporting, or dissemination of our research. All methods were performed in accordance with the relevant guidelines and regulations.

### Study design

The current study was designed as prospective specimen collection and retrospective blinded evaluation. From July 2015 to February 2016, we prospectively collected fecal samples at baseline from the patients with HBeAg positive CHB who would have oral antiviral therapy. Data analyst was blinded to clinical outcome of the patients and design of the study. Enrolled patients were randomly divided into training set and test set. We identified microbial signature and constructed classifier for HBeAg seroconversion using training set while validated the results using test set. Three groups were included in this survey: Group H, healthy participants; Group P, who remained HBeAg positive after more than 156 weeks of ETV- or TDF-based therapy; and Group N, who achieved HBeAg seroconversion at 12–156 weeks after ETV- or TDF-based therapy. Patients were ruled out from further analysis in the following cases, for example, who achieved HBeAg clearance while have not anti-HBeAg positive after more than 156 weeks of ETV- or TDF-based therapy. The study design and the construction of prediction model were shown in Supplementary Fig. 1.

### Study population

Eligible subjects were 18–50-year-old patients with HBeAg-positive CHB, who were anti-HBeAg negative before oral antiviral therapy and were undergoing ETV- or TDF-based antiviral therapy when they were recruited. Other eligibility criteria included: 18.5 ≤ body mass index (BMI) < 27.0; achieved HBeAg seroconversion that occurred at 12–156 weeks after ETV- or TDF-based therapy or remained HBeAg positive after more than 156 weeks of ETV- or TDF-based therapy; international normalized ratio ≤ 1.5. The exclusion criteria for the study were: alcoholism; presence of genetic or metabolic liver diseases, such as Wilson’s disease and hemachromatosis; co-infection with hepatitis C virus, hepatitis D virus, human immunodeficiency virus, Epstein–Barr virus, or cytomegalovirus; concurrent malignant tumors; autoimmune liver diseases; liver cirrhosis; moderate or severe nonalcoholic fatty liver disease or non-alcoholic steatohepatitis confirmed by ultrasound, computed tomography, or FibroScan; pregnant or lactating women; concurrent conditions such as diabetes mellitus, hypertension with blood pressure ≥ 160/100 mmHg, heart failure, and renal impairment; history of acute gastroenteritis within the last 8 weeks before enrollment. Patients who had a history of other antiviral treatment such as interferon or pegylated interferon, lamivudine, adefovir, or telbivudine were excluded. Patients who reported previous usage of antibiotics, and probiotics within the last 8 weeks were also excluded. Liver cirrhosis or hepatocellular carcinoma (HCC) was diagnosed based on comprehensive integration of clinical symptoms and physical signs, laboratory tests and medical imaging such as ultrasound, computed tomography, magnetic resonance imaging, and liver stiffness measurement, according to widely accepted guidelines. The diagnosis of HCC was further confirmed by histopathological examination. The detailed eligibility and exclusion criteria for the study are provided in Supplementary Table 1.

### Assessment of HBV markers and clinical outcomes

HBV markers were determined by several methods. For the HBsAg measurement, detection kit from Boson Biotech (Xiamen, Fujian, China; lower limit of detection of 0.1 IU/mL) was used. For the HBeAg and anti-HBeAg measurement, detection kit from Boson Biotech (Xiamen, Fujian, China) was used. The primary clinical outcome assessed in this study was HBeAg seroconversion, which was defined as HBeAg clearance (< 1.0 S/CO) along with positive anti-HBeAg. HBeAg and anti-HBeAg were doubly verified using a qualitative kit from InTec Products (Xiamen, Fujian, China). The status of HBeAg and anti-HBeAg was repeatedly determined with an interval of 12 weeks. If there were inconsistent in the two times of tests of HBeAg and anti-HBeAg, the observation time was extended until the results at two time points were consistent. The level of HBV DNA was determined by polymerase chain reaction (PCR) using a kit from Sansure Biotech (Changsha, Hunan, China), with a detection limit of 200 IU/mL. All laboratory tests were performed at the Clinical Laboratory, Zhongshan Hospital Affiliated to Xiamen University.

### Assessment and analysis of microbiota

Gut microbiota profiling was conducted by extracting genomic DNA from the stool of participants using the TIANamp Bacteria DNA Kit (TIANGEN, Haidian, Beijing, China). Microbial Sequencing was performed by Xiamen Anjie Medical Data Technology Co., Ltd. The 16S rDNA sequencing was performed on Illumina Hiseq platform. Ambiguous nucleotides, barcode and primer errors, homopolymeric nucleotides, and chimeras were filtered. FLASH was used for DNA assembling and quality control. The OUT is defined based on the similarity threshold of 97%. The OTU clustering was run using QIIME. The rarefaction curve was used to determine whether the sequencing depth was sufficient, which was run on QIIME. After genomic DNA extraction, the gene encoding for the V4 region of the 16S ribosomal DNA was amplified by PCR using universal primers. The universal primer pair contained index sequence and adaptor compatible for sequencing on a HiSeq2500 PE250 platform (Illumina, San Diego, California, USA). Through sequencing we obtained up to 70, 453 clean reads of fecal microbiota per sample (median = 46, 572). Prevalence of each genus from the gut microbiota signature for HBeAg seroconversion was estimated in both the subjects with and without HBeAg seroconversion.

### Metabolic profiling and phenotyping

Metabolic profiling was acquired using UHPLC-QTOF-MS (Agilent, Santa Clara, California, USA). First of all, metabolites were detected under both positive ion (POS) mode and negative ion (NEG) mode through relative standard deviation de-noising method. Half of the minimum value was used to fill up the missing values of raw data. Internal standard normalization method was used in this analysis. In order to perform principal component analysis (PCA), and orthogonal projections to latent structures-discriminate analysis (OPLS-DA), three-dimensional data involving the peak number, sample name, and normalized peak area were imported into SIMCA14.1 software package (V14.1, Sartorius Stedim Data Analytics AB, Umea, Sweden, https://landing.umetrics.com/downloads-simca). Metabolomics analysis was performed using the statistical correlation include the false discovery rate (FDR) correction. Figure based on PCA showed the distribution of origin data. In order to obtain a higher level of group separation and get a better understanding of variables responsible for classification, supervised OPLS-DA were applied. Seven-fold cross validation was used to estimate the robustness and the predictive ability of our model, such permutation test was proceeded in order to further validate the model. The R and Q intercept values were calculated after 200 permutations. The robustness of the models was measured by the low values of Q intercept, which had a low risk of over fitting and was reliable. A loading plot was constructed based on OPLS-DA, which was employed to show the contribution of variables to difference between two groups. The loading plot was also used to the important variables situated far from the origin. However, in case of many variables, the loading plot would be complex. The first principal component of variable importance in the projection (VIP) was obtained to refine this analysis. Metabolites with VIP values exceeding 1 were first selected as the changed metabolites. In step 2, the remaining variables were then assessed by Student’s t-test (*P* > 0.05), variables were discarded between two comparison groups. In addition, commercial databases including KEGG http://www.genome.jp/kegg/ and MetaboAnalyst http://www.metaboanalyst.ca/ were utilized to search for the pathways of metabolites^12,13^.

### Correlation between metabolic profiling and HBeAg seroconversion

For each set of comparisons, Euclidean distance matrix for the quantitative values of the differentiated metabolites was calculated. The differentiated metabolites were clustered by using complete linkage method and was displayed as a heatmap of hierarchical clustering analysis. Using KEGG database, differentiated metabolites were mapped to several specific pathways. The results of the metabolic pathway analysis were shown in a bubble plot.

### Correlation between microbiota and metabolic profiling

The correlation of differentiated metabolites between Group P and Group N was calculated by using the “spearman” algorithm. Correlation coefficient (Corr) was stored. Intestinal flora profiling with different abundance at genus level was acquired for subsequent association analysis. Heatmap was employed to indicate the correlation analysis between intestinal flora profiling and metabolic profiling with red indicated a Corr of 1 while blue indicated a Corr equaled to − 1. Data of the Corr with statistical difference were marked with “*” in the graph. Figure of correlation network was constructed by using Cytoscape_v3.7.1 software^14^.

### Statistical analysis

For microbiota profiling, one-way analysis of variance (ANOVA) was used to compare the means of relative abundance of bacterial phylum between the three groups. Least significant difference (LSD) method was applied to conduct multiple comparisons between pairs of groups. Diversity within samples, α-Diversity, was assessed by the richness as measured by number of operational taxonomic units (OTUs, 97% identity) at the same sequencing depth using the vegan R package. Diversity between samples, β-Diversity, was assessed by UniFrac distance, as well as the Bray–Curtis distance using Phyloseq R package^15^. Beta diversity between samples was also assessed by PCA and non-metric multidimensional scaling based on Hellinger transformation of relative abundance of all OTUs. One-way ANOVA was used to compare the diversity between the three groups. Microbiota clustering, enterotype stratification, was performed in fecal samples from CHB patients and healthy subjects using the Dirichlet multinomial mixture model^16^.

For microbial signature, univariate Wilcoxon test was used to select the original significant genera set between Group N and Group P. Random forest was implemented to select the least number of genera that distinguish Group N and Group P with appropriate accuracy on test set. Robustness of this method was validated by randomly splitting data into training and test sets. The area under the curve (AUC) was used to assess the discriminative ability of this prediction model.

To evaluate the robustness of the relationship between the microbial signatures for HBeAg seroconversion and other clinical parameters, we investigated the relative abundance of significant genera with clinical data in a co-inertia analysis. Relative abundance of microbial signature was transformed by Hellinger transformation, and clinical parameters including age, BMI, HBV DNA level, ALT and AST levels, and time to HBeAg seroconversion were transformed by logarithm function.

PCA was used to explore distribution of significant genera among all the genera and to analyze the relationship between HBeAg seroconversion, clinical data, and microbial signature. The relationship between variables was assessed using Spearman correlation test for continuous variables [e.g., principle component score and clinical data such as age, BMI, and levels of alanine aminotransferase (ALT), aspartate aminotransferase (AST), and HBV DNA]. Network between microbial signatures was plotted among Group N and Group P. For KEGG analysis, significant correlations between pathways of interest and microbial signature were detected to find the genetic cause.

## Results

### Clinical characteristics of CHB patients

In the enrolled patients, 37 of them achieved HBeAg seroconversion within 156 weeks after the initiation of oral antiviral therapy, while 41 subjects remained HBeAg positive even after over 156 weeks of therapy. Detailed clinical and demographic characteristics of the enrolled patients with CHB with or without HBeAg seroconversion (i.e., Groups N and P, respectively) are summarized in Supplementary Table 2. No differences were observed between Groups N and P with regards to body mass index and antiviral choices. Compared with the patients of Group P, the Group N individuals tended to have lower baseline age and HBV DNA, while higher baseline ALT and AST levels. More women were observed to fall in Group N than in Group P.

### Microbiota diversity in fecal samples

Regarding the phylum relative abundance, ANOVA indicated no significant difference between the main bacteria in Group N and Group P. *Bacteroidetes* and *Firmicutes* were the phyla with the most abundance in three groups (Supplementary Fig. 2A). Compared with healthy subjects (Group H), no significant difference was observed in the numbers of OTUs in Groups N and P (Supplementary Fig. 2B). Classical methods of β-diversity such as principal component analysis and Non-Metric Multi-Dimensional Scaling on relative abundance of all OTUs could not find significant differences between Groups H, N, and P (Supplementary Fig. 3).

### Enterotype clustering in CHB patients and healthy subjects

**Figure 1:**
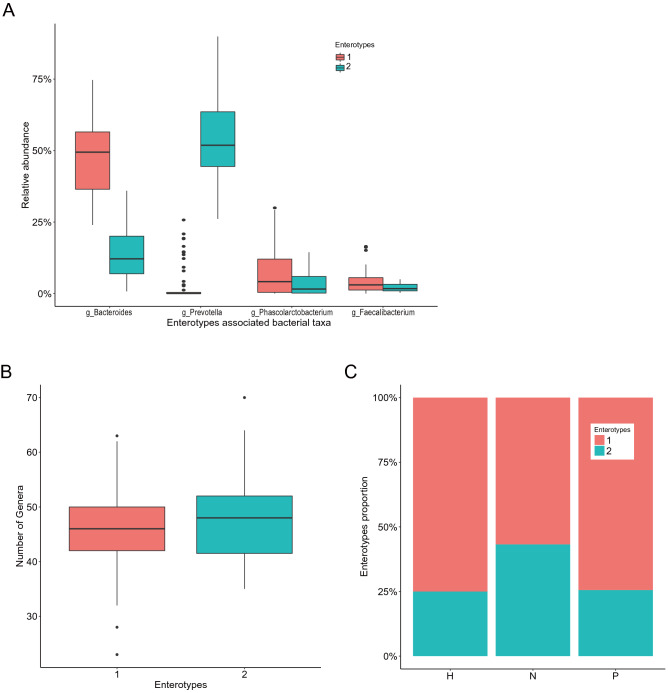
Enterotype clustering in the patients with CHB and healthy controls. (**A**) Two distinct microbiota communities are clustered with one enterotype is enriched in *Bacteroides* (50% of samples) while the other one is enriched in *Prevotella* (52% of samples). (**B**) *Prevotella* enterotyped subjects harbor higher diversity, as compared with *Bacteroides* enterotyped subjects. (**C**) Group N has higher proportion of *Prevotella* enterotyped subjects than that in Group P.

Microbiota was found to separate optimally into two distinct microbiota communities as assessed by Laplace parameter and Akaike information criterion (Supplementary Fig. 4). One enterotype was enriched in *Bacteroides* (50% of samples) while the other one was enriched in *Prevotella* (52% of samples) (Fig. 1A). Notably, *Prevotella* enterotyped subjects harbored higher diversity, as compared with *Bacteroides* enterotyped subjects (Fig. 1B). In Group N, the proportion of *Prevotella* enterotyped subjects was higher than that in Group P (Fig. 1C).

### Identification of the microbial signature for HBeAg seroconversion

**Figure 2:**
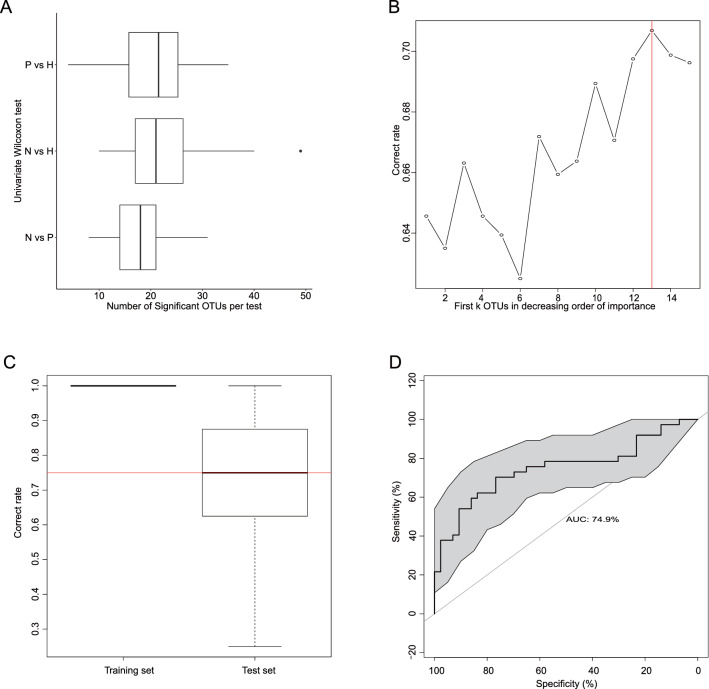
Univariate comparison and machine learning based on HBeAg seroconversion. (**A**) Wilcoxon test is conducted to identify the significant genera in paired groups (P vs. H, N vs. H, N vs. P). This procedure is repeated 100 times on bootstrapped sample set. About 18–22 genera are selected. The number of significant genera for each comparison is reported with boxplot. (**B**) Thirteen genera are selected as microbial signature for HBeAg seroconversion to guarantee about 71% accuracy rate on test data. (**C**) The mean of accuracy rate on test data is about 0.75, indicating this method is robust. (**D**) The area under the ROC curve (AUC) is 74.9%, which indicates the prediction model has a significant discriminative ability.

We further explored the association between HBeAg seroconversion and fecal microbiota composition. Wilcoxon test was conducted to identify the significant genera in paired groups (P vs. H, N vs. H, and N vs. P), and this procedure was repeated 100 times on bootstrapped sample sets. About 18 (median) genera could discriminate Group N from Group P, while about 21 (median) genera could distinguish Group N from Group H, and 22 (median) genera distinguished Group P from Group H (Fig. 2A). Wilcoxon test identified microbial signatures between Groups N and P. However, this univariate analysis omitted the correlation between genera and gave a set with a large number of significant genera.

We then tested the random forest method for identification of the microbial signature. Firstly, Wilcoxon test was used to identify 18 genera that showed significant differences between Groups N and P. Then we modelled the HBeAg seroconversion and these significant genera with random forest; the relative importance measured for each genus was given in a descending order. Secondly, we separately assessed the classification accuracy of the first k (k = 1, 2, 3, …, 13, 14, 15) genera. We randomly split all the data into training and test sets in a 3:1 ratio, predicted the HBeAg seroconversion with first k genera on the training set and then validated the model on test set. The process was repeated 100 times to calculate the mean classification accuracy rate on test data. In this step, 13 genera were selected as microbial signature for HBeAg seroconversion to guarantee about 71% accuracy rate on test data (Fig. 2B). The number of significant genera selected by random forest was far less than that obtained by Wilcoxon test, indicating the better performance of variable selection by the random forest method. Boxplot of correct classification rate on training data and test data was also given by 100 times repetition of the calculation at second step. The mean of the accuracy rate on test data was lower than that on training data, and the corresponding standard deviation was higher than that obtained with the training data (Fig. 2C). The distribution of the accuracy rate on test data suggested that this method is robust. Thirdly, receiver operating characteristic was obtained for the evaluation of the classification model based on microbial signature. The area under the curve (AUC) was 74.9%, which indicated a significant discriminative ability (Fig. 2D). Finally, we assessed the classification accuracy with different sample size based on repeat sampling. With increasing sample size, the mean classification accuracy rate ranged from 67 to 72%, so we concluded that our sample size was adequate (Supplementary Fig. 5).

### Taxonomy of the Gut microbial signature for HBeAg seroconversion

**Figure 3:**
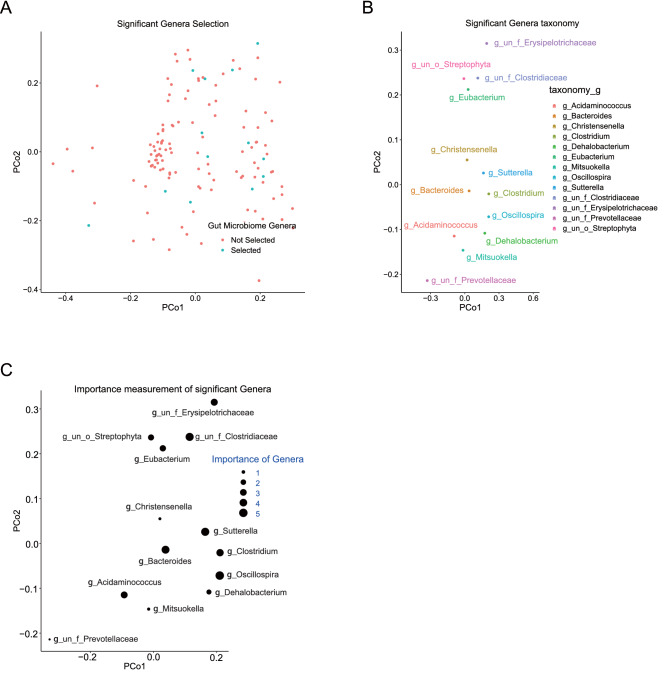
Taxonomic assessment of genus microbiota signature for HBeAg seroconversion. Axes represent the two first components from principal coordinate analysis (PCoA) based on the phylogenetic distance between genus representative sequences. (**A**) Thirteen genera (blue dots) are selected out from 169 genera using random forest while red dots represent unselected genera. (**B**) Taxonomic assignation of the significant genera at genus level. (**C**) The importance of significant genera is sized by the respective dot.

Using random forest, 13 genera were screened out as potential microbial signature from the total 169 genera of Group N and Group P (Fig. 3A). Using Kulczynski distance between the representative sequences of all genera, we performed principal component analysis to assess the distribution of the microbial signature for HBeAg seroconversion. As shown in Fig. 3B, the microbial signature was as taxonomically diverse as the genera from the whole microbiota data set. The microbial signature for HBeAg seroconversion was characterized and the dominant genera were identified to be *Bacteroides*,* Sutterella*, and *Oscillospira* (Fig. 3B). The 13 genera for HBeAg seroconversion were ranked according to their importance in the model (Supplementary Table 3 and Supplementary Fig. 6). In this model, genera with more importance usually had positive association with HBeAg seroconversion. The importance of the genera was shown in Fig. 3C.

### Gut microbial signature for HBeAg seroconversion and association with clinical and microbial parameters

**Figure 4:**
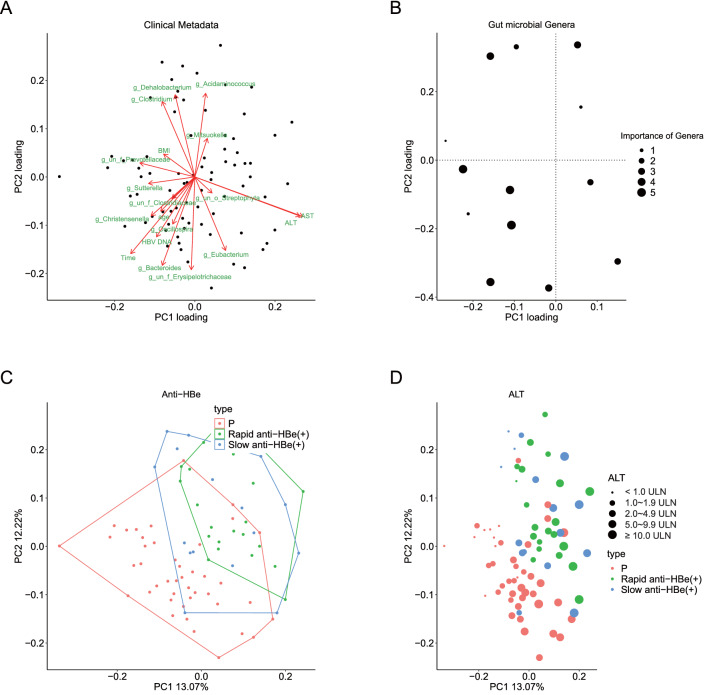
Interactions between clinical, microbial ecology parameter and microbial signatures for HBeAg seroconversion. A co-inertia analysis is performed between the relative abundance of microbial signature genera for HBeAg seroconversion and clinical parameters. (**A**) Scatter plot of the first two PC loadings of clinical data. Each clinical parameter and microbial signature are labelled in red. (**B**) Scatter plot of the first two PC loadings for the importance of microbial signatures. Size accounts for the importance. (**C**,**D**) Scatter plot of the first two components of co-inertia analysis. Each dot represents a fecal microbiota sample. Orange dots represent the subjects that remained HBeAg positive even after more than 3 years of oral antiviral therapy (Group P), while green dots represent the subjects that achieved HBeAg seroconversion in less than 12 months after the initiation of oral antiviral therapy [Rapid anti-HBe (+)], and blue dots represent the subjects that achieved HBeAg seroconversion later than 12 months after the initiation of oral antiviral therapy [Slow anti-HBe (+)]. (**C**) Using random forest based on the microbial signature and clinical data of Group P and Group N patients, rapid anti-HBe (+) and Group P are well separated. (**D**) Baseline ALT has a positive relation with HBeAg seroconversion. Size represents the value of ALT.

The first 2 co-inertia principle components (PCs) could explain about 25.3% of co-variation. An undefined *Prevotellaceae*, *un_f_Prevotellaceae*, (PC1 loading − 0.27) and *Genus Sutterella* (PC1 loading − 0.22) were confirmed to be the most important parameters contributing to variation along the first co-inertia component (PC1), while undefined *Erysipelotrichaceae*, *un_f_Erysipelotrichaceae* (PC2 loading − 0.37) and *Genus Bacteroides* (PC2 loading − 0.36) were the most important parameters along the second co-inertia component (PC2) (Fig. 4A). The genera that had higher importance in the microbial signature were negatively associated with PC1 (Fig. 4B). Group N showed tendency to have positive PC1 score, while Group P tended to have negative PC1 score (Fig. 4C). Loadings of microbial signatures and clinical parameters along the PC1 and the PC2 were shown in Supplementary Table 4. PC1 was negatively associated with time to HBeAg seroconversion (rho = − 0.5108, *P* < 0.05) (Supplementary Fig. 7A). No significant association was observed between HBV DNA and PC2 (Supplementary Fig. 7B). PC1 was positively associated with ALT (rho = 0.8060, *P* < 0.05) and AST (rho = 0.8354, *P* < 0.05) levels (Fig. 4D and Supplementary Fig. 7C, respectively). *Genera un_f_Prevotellaceae* and *un_f_Erysipelotrichaceae* were shown in Supplementary Fig. 8A,B, respectively, while *Genera Sutterella* and *Bacteroides* were shown in Supplementary Fig. 8C,D, respectively. Additionally, age is positively associated with time to HBeAg seroconversion (rho = 0.2664, *P* < 0.05). Correlations between microbial signatures and clinical parameters were also shown in Supplementary Table 5.

### Network of reciprocal interaction

We separately calculated the correlation network between 13 significant genera with large relative abundance in microbial signatures among Groups N and P. The measure of correlation used was the Spearman Correlation coefficient, and only significant correlations were included in the network (Supplementary Fig. 9). In Group N, significant positive association was observed between *Genus Dehalobacterium* and *Genus Christensenella*. Pronounced positive association was also observed between *Genus Clostridium* and *Genus un_f_Clostridiaceae*. However, negative association existed between *Genus Eubacterium* and *Genus Acidaminococcus*. In Group P, significant positive association was observed between *Genus Dehalobacterium* and *Genus Eubacterium*, *Genus Clostridium* and *Genus un_f_Prevotellaceae*, *Genus Bacteroides* and *Genus Sutterella*. On the contrary, negative association existed between *Genus Dehalobacterium* and *Genus Bacteroides*, *Genus Dehalobacterium* and *Genus Sutterella*.

### Correlation between metabolic profiling and HBeAg seroconversion

**Figure 5:**
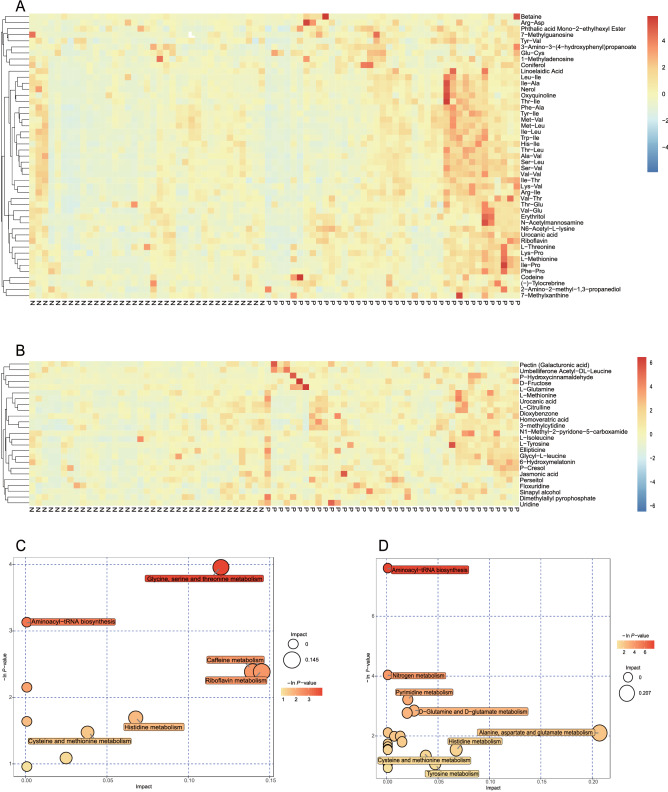
Correlation between differentiated metabolites and HBeAg seroconversion. The differentiated metabolites are defined as *P* < 0.05 (Student's t-test), combined with variable importance in the projection of the first principal component is greater than 1 in the model of orthogonal projections to latent structures-discriminant analysis. The differentiated metabolites are clustered by using complete linkage method and are displayed as a heatmap of hierarchical clustering analysis with the abscissa represents the different experimental cohorts while the ordinate represents the differentiated metabolites. The color blocks at different positions represent the relative expression of the specific metabolites at the corresponding positions. (**A**) Heatmap of hierarchical clustering analysis under positive ion (POS) mode. (**B**) Heatmap of hierarchical clustering analysis under negative ion (NEG) mode. Using KEGG database, differentiated metabolites are mapped to related metabolic pathways, which are shown in a bubble plot. Each bubble in the bubble diagram represents a metabolic pathway. Size of bubble indicates the influence factor of a specific metabolic pathway in topological analysis. The size is larger when the influence factor is higher. The ordinate and the color of bubble indicate the enrichment analysis. The bubble is darker when P value (expressed in minus natural logarithm, i.e., − ln *P*-value) is more significant in analysis. (**C**) Enrichment analysis of metabolic pathways under positive ion (POS) mode. (**D**) Enrichment analysis of metabolic pathways under negative ion (NEG) mode.

A total of 1831 metabolites were detected under positive ion mode while 1636 metabolites were detected under negative ion mode. Significant difference regarding the intestinal metabolites was observed between and Group P and Group N. Group P tended to have higher abundance of some specific metabolites such as Betaine, essential amino acids, and several dipeptides in fecal samples under both positive ion mode and negative ion mode (Fig. 5A,B). Using KEGG database, differentiated metabolites could be mapped to the following pathways: Aminoacyl-tRNA biosynthesis, and Glycine, serine and threonine metabolism (Fig. 5C,D).

### Correlation between microbiota and metabolic profiling

Different metabolic profiling between group N and group P were shown by OPLS-DA (Supplementary Fig. 10). Correlation analysis indicated that *Genus Christensenella* can explain most of the differentiated metabolites in fecal samples between Group P and Group N under both positive ion mode and negative ion mode (Supplementary Fig. 11A,B). Correlation network analysis also showed that *Genus Christensenella* was closely related to most of the differentiated metabolites (Supplementary Fig. 11C,D).

## Discussion

In the present study we characterized the signature of fecal microbiota in a cohort of CHB patients with or without HBeAg seroconversion. Using a machine learning approach, we showed that HBeAg seroconversion is associated with a distinct fecal microbiota signature, which could also be associated with ALT and AST levels. We explored the data using a combination of classical approaches for microbiota analysis. However, these yielded no pronounced differences between the CHB patients with or without HBeAg seroconversion. Group N tended to have higher abundance of *Firmicutes* phylum and a lower abundance of *Bacteroidetes* phylum, although no significant difference was detected when compared with Group P.

In the present study, we further explored our data using Wilcoxon test and random forest that identified a robust microbial signature. At the family level, the microbial signature that was enriched included *Bacteroidaceae*, *Alcaligenaceae*, and *Ruminococcaceae*. *Genus Un_f_Prevotellaceae* and *Genus Sutterella* displayed negative association with PC1. This indicates that patients with higher abundance of *Un_f_Prevotellaceae* and *Genus Sutterella* have less opportunities to get HBeAg seroconversion. On the other hand, *Genus un_f_Erysipelotrichaceae* and *Genus Bacteroides* were the most important factors associated with second co-inertia component and were negatively associated with HBeAg seroconversion.

Several algorithms have been developed to predict the efficacy of interferon-based antiviral therapy for CHB^17–19^. However, no such algorithms have been reported for testing the efficacy of nucleos(t)ide analogues in the treatment of CHB. Since intestinal microbiota has a critical or even decisive role on the outcome of chronic HBV infection, it seems very likely that Group N would have different microbial signatures from those of Group P, which can serve as the base of a novel prediction model. Using classical ecologic approaches, we found considerable overlap between Groups N and P, with no significant differences. However, when random forest method was applied, Group N could be well distinguished from Group P. Notably, the AUC area of the established prediction model for the test set was 0.749, indicating a robust model that can predict whether HBeAg seroconversion is likely to occur in a specific individual.

In recent years, several studies have reported gut dysbiosis in patients with CHB^20,21^. Besides CHB, gut dysbiosis also plays a critical role in liver fibrosis and the genesis of hepatocellular carcinoma related to non-alcoholic fatty liver disease^22,23^. Chen et al.^24^ have characterized the alteration of *Bacteroidetes* phylum in case of HBV or alcohol-related cirrhosis. However, the relative abundance of *Bacteroidetes* in the patients with HBeAg seroconversion versus without seroconversion remains unconfirmed. Our study shows that there is differential abundance of *Firmicutes* and *Bacteroidetes* phylum in the patients with or without HBeAg seroconversion. *Bacteroides* is the most prevalent Gram-negative gut bacterial taxon, and is the main source of lipopolysaccharide (LPS)^25^. Pathogen-derived LPS is a potent ligand for host receptor toll-like receptor 4 (TLR4), which plays an important role in sensing bacteria as part of the innate immune response. Research by Chou et al.^9^ suggested that LPS-TLR4 signaling plays a critical role in the immune tolerance to HBV infection in young mice. After binding to its receptor TLR4, LPS triggers the M1 or M2 polarization of Kupffer cells. M1 polarization leads to the clearance of HBV, while M2 polarization of Kupffer cells support HBV persistence by suppressing HBV-specific CD8^+^ T cells^26^. Elderly individuals usually have higher abundance of *Bacteroidetes*^27^, which is linked to elevated levels of serum LPS. Thus, it seems reasonable that the elderly tends to have poorer efficacy of antiviral treatment, which is consistent with clinical observations. For both HBeAg-positive and HBeAg-negative patients, younger age (< 30 years) was a significant predictor of ALT normalization after telbivudine treatment^28^. More than that, age was one of the significant predictive factors for sustained virologic response^29^. After HBeAg seroconversion, patients aged > 40 relapsed more frequently than those aged ≤ 40^30^. Therefore, higher abundance of *Bacteroidetes* may, at least partly, explain the lower likelihood of HBeAg seroconversion in older patients.

As is shown in Fig. 5 and Supplementary Fig. 11, patients who achieved HBeAg seroconversion tended to have lower abundance of certain fecal metabolites such as essential amino acids, and several dipeptides, which had positive correlation with the relative abundance of *Genus Christensenella*. *Genus Christensenella* is a kind of newly isolated gram-negative, strictly anaerobic bacterium that inhabits the human intestine. Recent finding indicates that *Genus Christensenella* can only trigger a weak inflammatory response through the NF-κB signalling pathway, which may partly explain why the patients in Group P with higher abundance of *Genus Christensenella* is unlikely to obtain HBeAg seroconversion^31^. Study has indicated there are certain relationship between plasma amino acid profiles and various stages of HBV infection^32^. However, no confirmed correlation between fecal amino acid profiles and HBV infection has been reported, which remains to be further elucidated. Amino acids may as metabolic mediators of the cross talk between host and pathogen^33^. According to KEGG database, fecal metabolites with different abundance such as essential amino acids and dipeptides could be mapped to several pathways: aminoacyl-tRNA biosynthesis, and glycine, serine and threonine metabolism. Recent discovery has revealed that cytoplasmic aminoacyl-tRNA synthetases can sense and respond to danger signals and induce antiviral immunity^34^. It seems reasonable that different intestinal flora affects the metabolism of amino acids which affects the activity of aminoacyl-tRNA synthetases, and further regulates the antiviral immunity of host against HBV.

A limitation of the present study is that no mucosal sample was obtained for microbiota analysis. Since there were no significant bowel changes in the CHB patients, the participants were usually not ready to accept colonoscopy examination. Secondly, for Group P and Group N, only about 40 cases were enrolled in each group. Nonetheless, according to statistical inference, this sample size does not significantly impair the accuracy of the prediction model. As can be seen from Supplementary Fig. 5, if the total number of Group N and Group P is less than 45, increasing the sample size can sharply improve the mean accuracy. However, if the sample size is more than 45, the mean accuracy rate increases slowly. It can be inferred that increasing sample size would have had a further but less significant improving effect on the mean accuracy. Thus, the sample size in our study is adequate.

To conclude, machine learning based random forest allowed us to identify gut microbial signature for HBeAg seroconversion, which also could be reproduced by the test set. Importantly, because of its relatively high accuracy, the prediction model based on the gut microbial signature can be used not only as a clinical predictor of HBeAg seroconversion before the initiation of oral antiviral therapy, but also as a way to explore the relevant genera for HBeAg seroconversion in the future. Our study highlights the difficulty in stratifying patients based on a microbiota profile when only classical ecologic approaches are employed. The use of machine learning has allowed us to better explore the microbial data, allowing us to identify certain links between gut microbiota and the clinical outcome.

## Supplementary Information

Supplementary Figure 1.

Supplementary Figure 2.

Supplementary Figure 3.

Supplementary Figure 4.

Supplementary Figure 5.

Supplementary Figure 6.

Supplementary Figure 7.

Supplementary Figure 8.

Supplementary Figure 9.

Supplementary Figure 10.

Supplementary Figure 11.

Supplementary Legends.

Supplementary Tables.

## Abbreviations

CHB: Chronic hepatitis B
HBV: Hepatitis B virus
TDF: Tenofovir disoproxil fumarate
HBeAg: HBV e-antigen
TLR4: Toll-like receptor 4
OTU: Operational taxonomic unit
AUC: Area under the curve
ALT: Alanine aminotransferase
AST: Aspartate aminotransferase
PC: Principal component
LPS: Lipopolysaccharide
