# Supplementary figures and images for: Meta-omics characteristics of intestinal microbiota associated to HBeAg seroconversion induced by oral antiviral therapy
Source: Sci Rep. 2021 Feb 5;11:3253. doi: 10.1038/s41598-021-82939-1 (PMC7864979; doi:10.1038/s41598-021-82939-1)

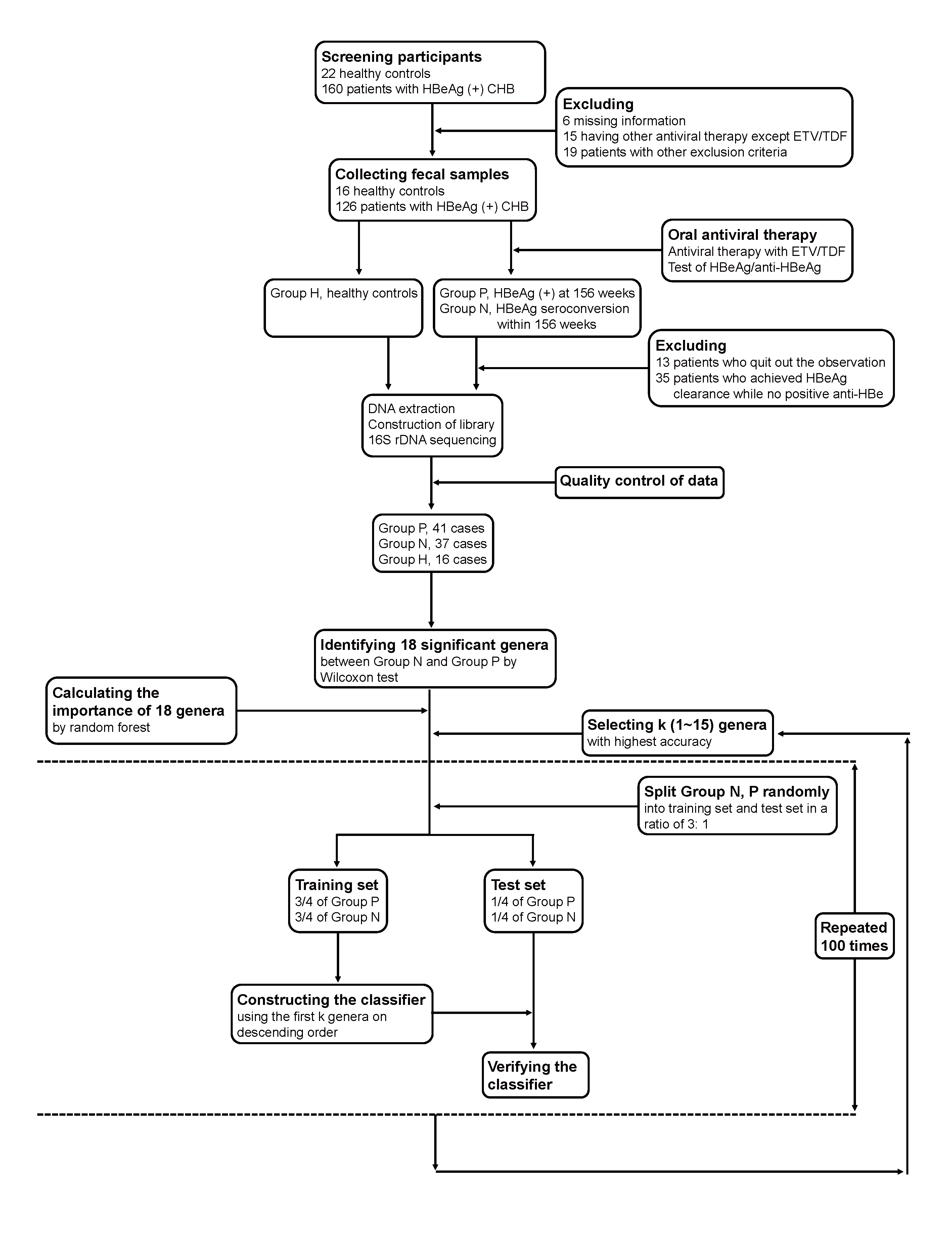

Supplement: Supplementary file 1 — Supplementary Figure 1. [file 41598_2021_82939_MOESM1_ESM.tiff]

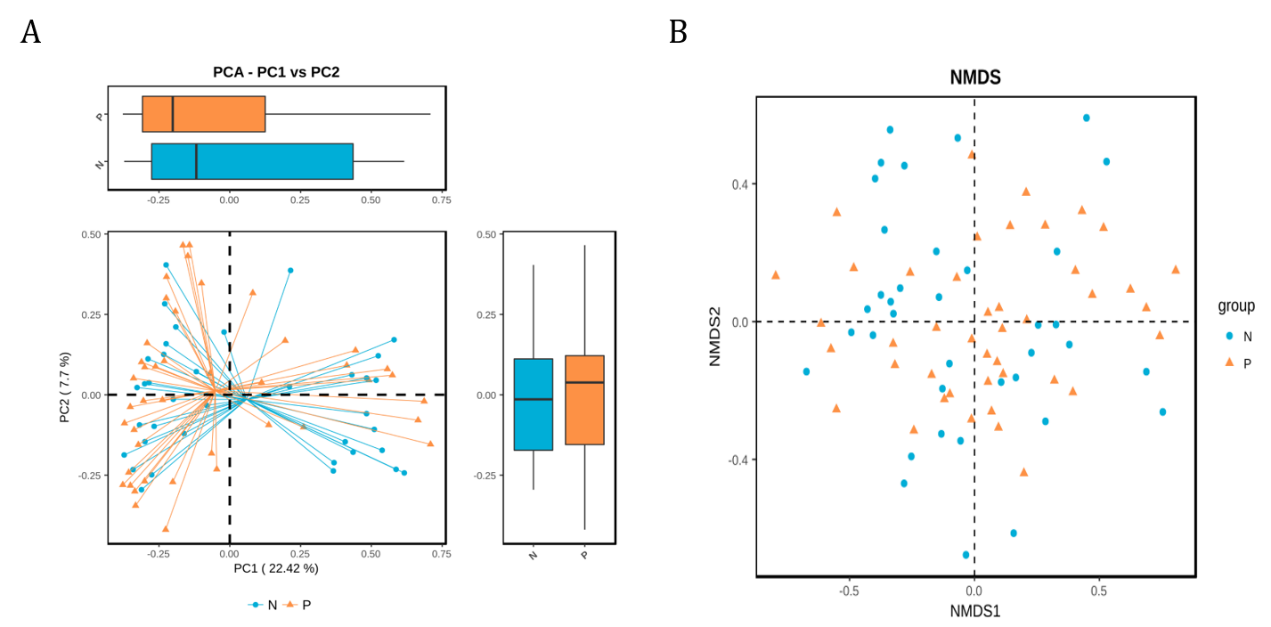

Supplement: Supplementary file 3 — Supplementary Figure 3. [file 41598_2021_82939_MOESM3_ESM.tif]

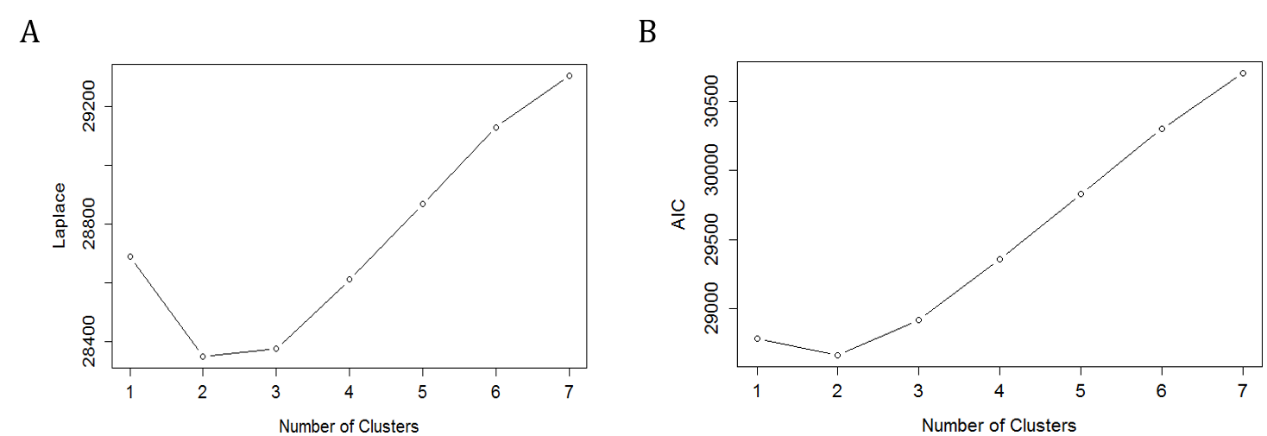

Supplement: Supplementary file 4 — Supplementary Figure 4. [file 41598_2021_82939_MOESM4_ESM.tif]

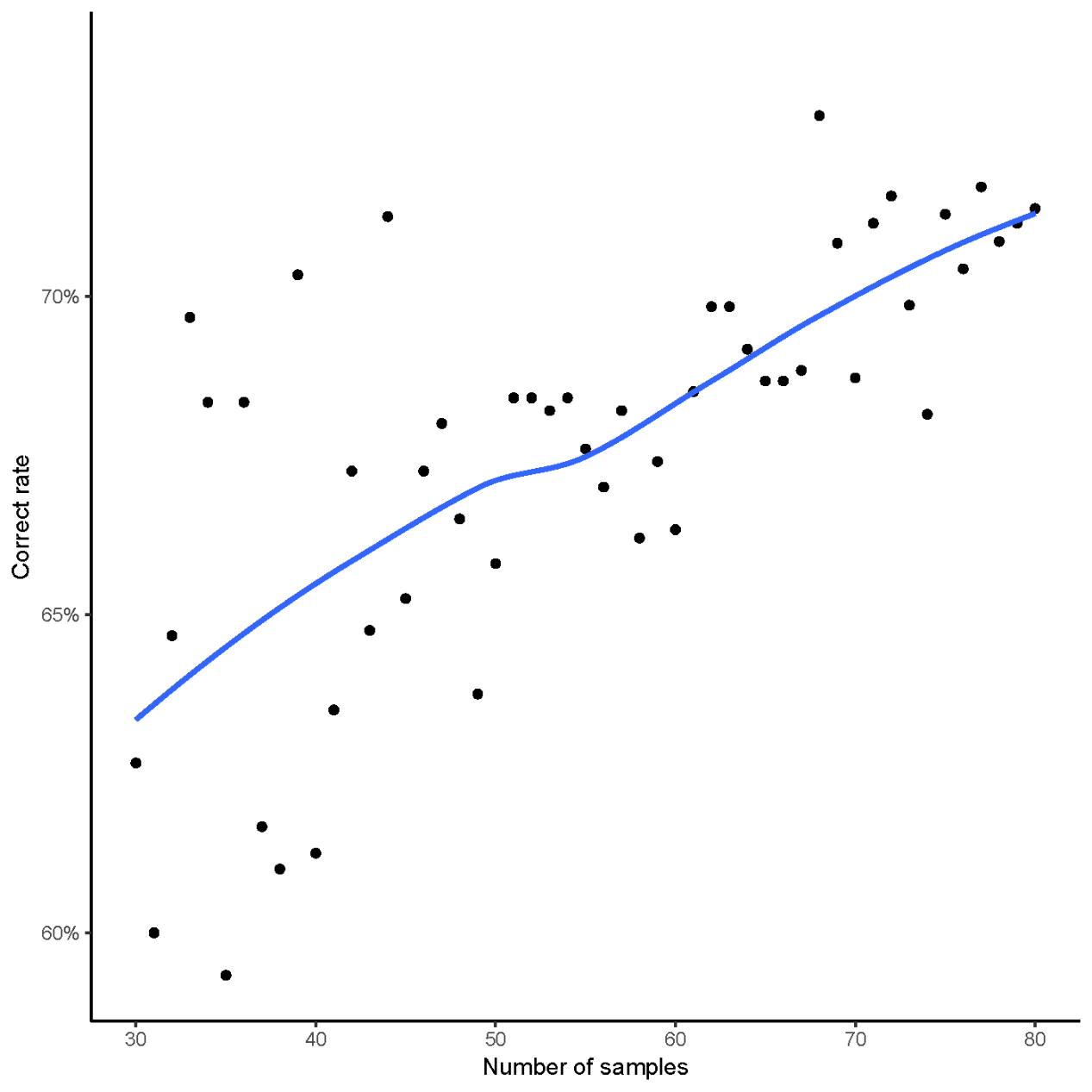

Supplement: Supplementary file 5 — Supplementary Figure 5. [file 41598_2021_82939_MOESM5_ESM.tif]

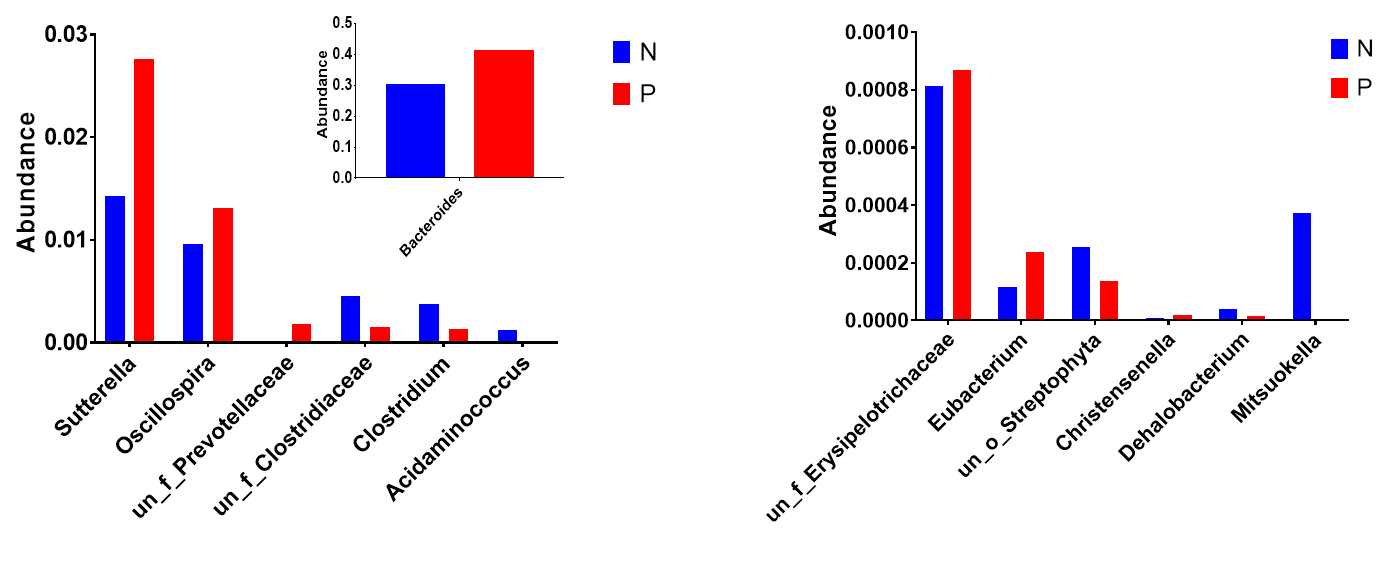

Supplement: Supplementary file 6 — Supplementary Figure 6. [file 41598_2021_82939_MOESM6_ESM.tif]

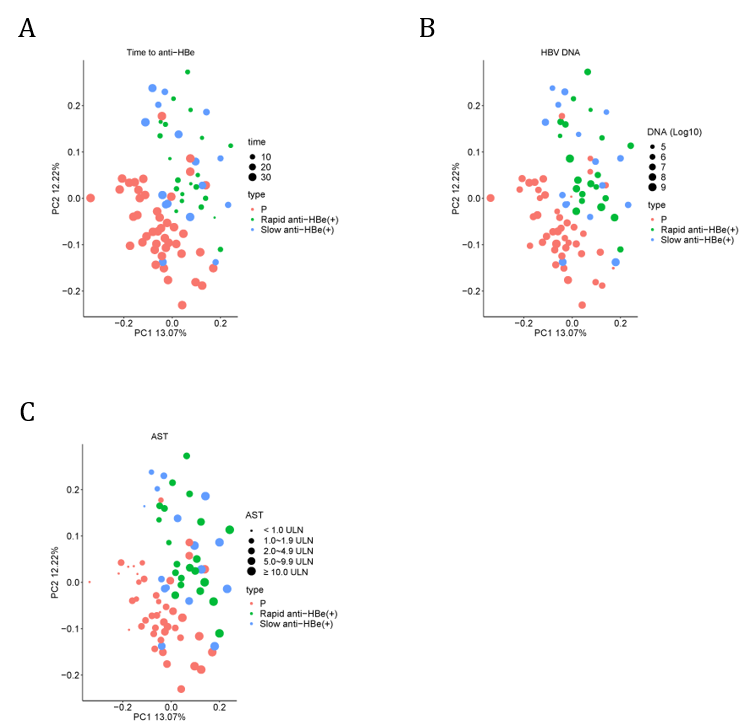

Supplement: Supplementary file 7 — Supplementary Figure 7. [file 41598_2021_82939_MOESM7_ESM.tif]

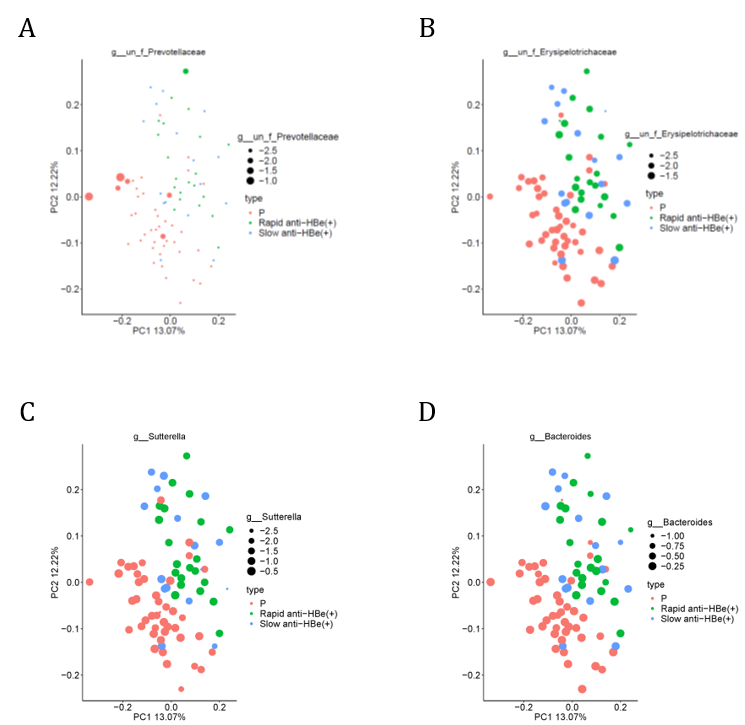

Supplement: Supplementary file 8 — Supplementary Figure 8. [file 41598_2021_82939_MOESM8_ESM.tif]

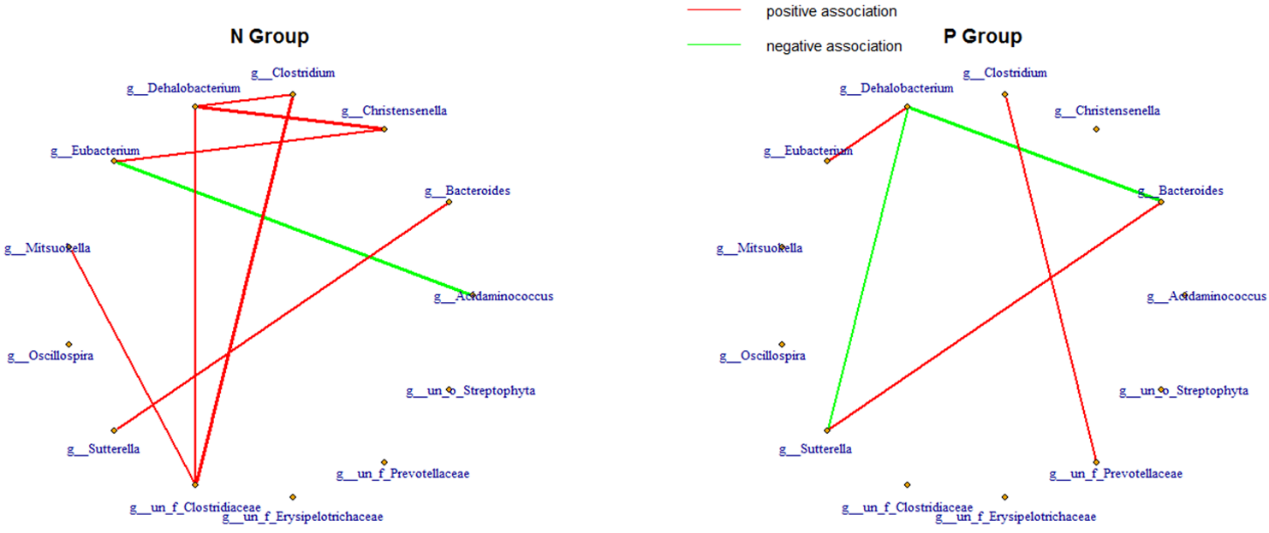

Supplement: Supplementary file 9 — Supplementary Figure 9. [file 41598_2021_82939_MOESM9_ESM.tif]

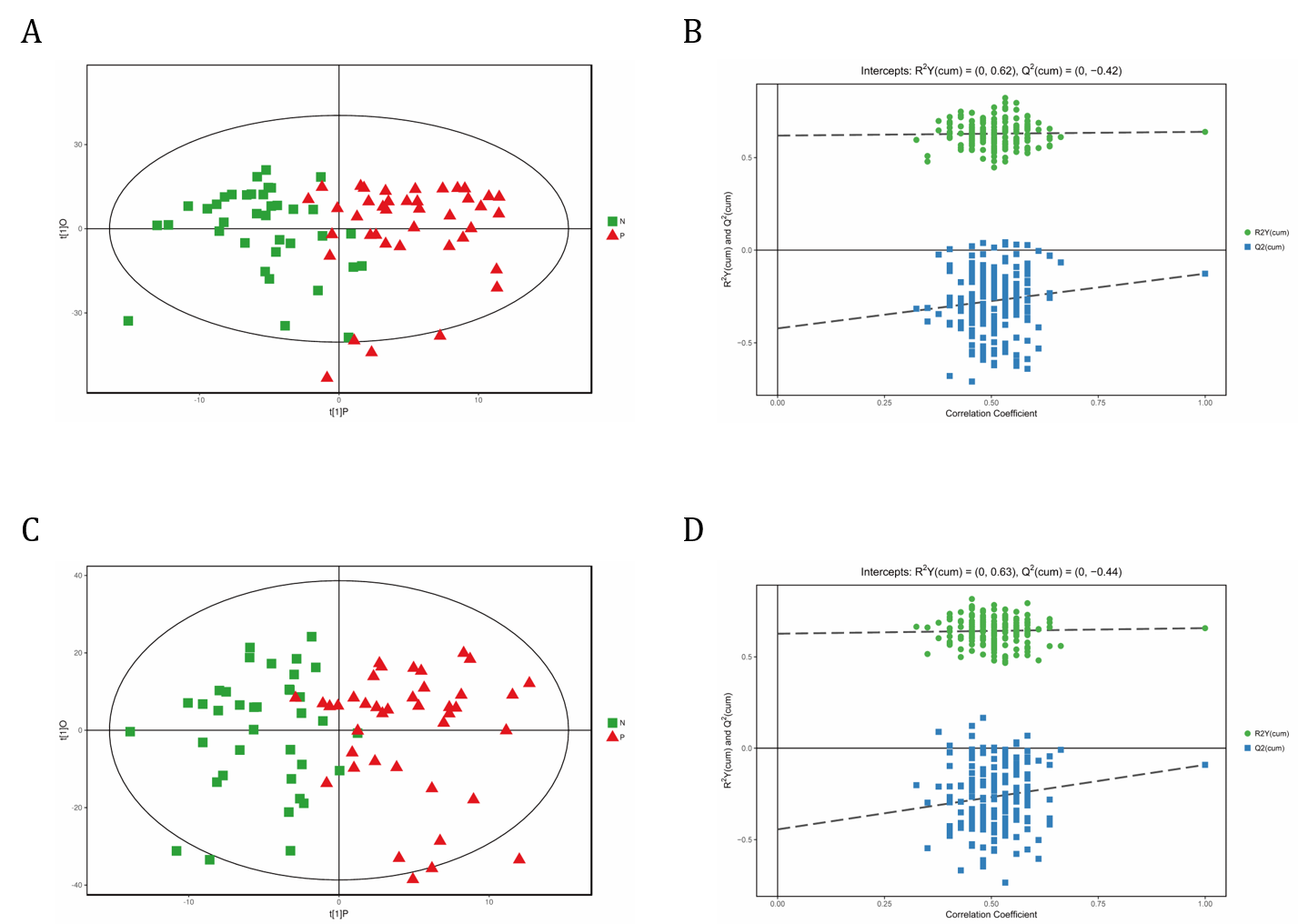

Supplement: Supplementary file 10 — Supplementary Figure 10. [file 41598_2021_82939_MOESM10_ESM.tif]
