# Supplementary material for: Meta-omics characteristics of intestinal microbiota associated to HBeAg seroconversion induced by oral antiviral therapy
Source: Sci Rep. 2021 Feb 5;11:3253. doi: 10.1038/s41598-021-82939-1 (PMC7864979; doi:10.1038/s41598-021-82939-1)
Supplement: Supplementary file 12 — Supplementary Legends. [file 41598_2021_82939_MOESM12_ESM.docx]

## Meta-Omics Characteristics of Intestinal Microbiota Associated to HBeAg Seroconversion Induced by Oral Antiviral Therapy

## Running title: Microbiota and HBeAg Seroconversion

Yu-Li Zeng, MD^1†^, Lei Qin, PhD^2†^, Wen-Jun Wei, MD^1†^, Hong Cai, MD^3^, Xiao-Fang Yu, MD^3^, Wei Zhang, MD^4^, Xiao-Lu Wu, MD^5^, Xiao-Bin Liu, MD^5^, Wei-Ming Chen, MD^1^, Pan You, MD, PhD^6^, Mei-Zhu Hong, MD^7^, Yaming Liu, MD, PhD^1^, Ben-Chang Shia, PhD^8*^, Jian-Jun Niu, MD, PhD^6*^ and Jin-Shui Pan, MD, PhD^9*^

^1^Department of Gastroenterology, Zhongshan Hospital Xiamen University, Xiamen, Fujian, China

^2^School of Statistics, University of International Business and Economics, Chaoyang District, Beijing, China

^3^Department of Hepatology, Xiamen Hospital of Traditional Chinese Medicine, Xiamen, Fujian, China

^4^Department of Research Institute, the Fifth Hospital of Shijiazhuang, Shijiazhuang, Hebei, China

^5^Department of Infectious Diseases, the First Affiliated Hospital of Xiamen University, Xiamen, Fujian, China

^6^Center of Clinical Laboratory, Zhongshan Hospital Xiamen University, Xiamen, Fujian, China

^7^Department of Traditional Chinese Medicine, Zhongshan Hospital Xiamen University, Xiamen, Fujian, China

^8^School of Management & Big Data Research Centre, Taipei Medical University, Taipei, Xinyi, Taiwan

^9^Liver Research Center, the First Affiliated Hospital of Fujian Medical University, Fuzhou, Fujian, China;

^†^These authors contributed equally.

^*^**Correspondence:**

Jin-Shui Pan, MD, PhD, E-mail: [j.s.pan76@gmail.com](mailto:j.s.pan76@gmail.com);

Jian-Jun Niu, MD, PhD, E-mail: [niujianjun62@163.com](mailto:niujianjun62@163.com);

Ben-Chang Shia, PhD, E-mail: [stat1001@tmu.edu.tw](mailto:stat1001@tmu.edu.tw)

## Supplementary Figure legends

Supplementary Figure 1**.**Study design and the construction of prediction model.

Fecal samples from 22 healthy controls (Group H) and 160 patients with HBeAg positive CHB are prospectively collected. Among them, 40 participants are excluded because of losing information or other exclusion criteria. After 156 weeks of oral antiviral therapy, 41 patients remain HBeAg positive (Group P) while 37 cases achieve HBeAg seroconversion (Group N). For participants in Group H, N, and P, 16S rDNA sequencing is performed. Eighteen significant OTUs are screened out by Wilcoxon test between Group N and Group P. The importance of 18 OTUs is calculated by random forest. The classification accuracy of the first k (k = 1 ~ 15) OTU is separately assessed. The participants in Group N and Group P are randomly divided into training set and test set at a ratio of 3:1. The training set is employed for the construction of classifier while the test set is used for the validation of classifier. The process is repeated 100 times to calculate the mean classification accuracy rate on test data.

Supplementary Figure 2. Taxonomic and diversity analysis of fecal microbiota in CHB patients with or without HBeAg seroconversion, and healthy subjects.

(A) Group N tends to have lower relative abundance of phylum *Bacteroidetes* while higher relative abundance of phylum *Firmicutes* in stool specimens. However, no statistical difference is observed (ANOVA, *P* = 0.4251 and 0.5806, respectively). (B) No significant differences are observed in the α-diversity measured by OTU number (Wilcoxon test, P = 0.7195).

Supplementary Figure 3. Beta diversity calculated by traditional methods cannot find differences in fecal microbiota between groups.

(A) No significant differences are observed between groups by principal component analysis (PCA). (B) No significant differences are observed between groups by Non-Metric Multi-Dimensional Scaling (NMDS).

Supplementary Figure 4. Two enterotypes are found upon application of Laplace parameter (A) and Akaike information criterion (AIC) (B).

Supplementary Figure 5. The classification accuracy under different sample size based on repeat sampling. With increasing sample size, the mean classification accuracy rate ranges from 67% to 72%.

Supplementary Figure 6. The abundance of significant genus between group N and group P.

Supplementary Figure 7. A co-inertia analysis is performed for the interactions between HBeAg seroconversion and clinical parameter. Orange dots represent the subjects that remained HBeAg positive even after more than 3 years of oral antiviral therapy (P), while green dots represent the subjects that achieved HBeAg seroconversion in less than 12 months after the initiation of oral antiviral therapy (Rapid anti-HBe (+)), and blue dots represent the subjects that achieve HBeAg seroconversion later than 12 months after the initiation of oral antiviral therapy (Slow anti-HBe (+)).

(A) Scatter plot of the first two PC loadings of the time taken to achieve HBeAg seroconversion. Size represents the time to achieve HBeAg seroconversion (month). (B) Scatter plot of the first two PC loadings of the interaction between baseline HBV DNA level and the achievement of HBeAg seroconversion. Size represents the level of baseline HBV DNA titer. (C) Baseline AST has a positive relation with HBeAg seroconversion. Size represents the value of baseline AST.

Supplementary Figure 8. A co-inertia analysis is performed for the interactions between HBeAg seroconversion and microbial ecology parameter. Orange dots represent the subjects that remained HBeAg positive even after more than 3 years of oral antiviral therapy (P), while green dots represent the subjects that achieved HBeAg seroconversion in less than 12 months after the initiation of oral antiviral therapy (Rapid anti-HBe (+)), and blue dots represent the subjects that achieve HBeAg seroconversion later than 12 months after the initiation of oral antiviral therapy (Slow anti-HBe (+)).

(A) The *Genus un_f_Prevotellaceae* has a negative association with PC1. Size represents the relative abundance of *Genus un_f_Prevotellaceae*. (B) The *Genus* *un_f_Erysipelotrichaceae* has a negative association with PC2. Size represents the relative abundance of *Genus* *un_f_Erysipelotrichaceae*. (C) The *Genus* *Sutterella* has a negative association with PC1. Size represents the relative abundance of *Genus* *Sutterella*. (D) The *Genus* *Bacteroides* has a negative association with PC2. Size represents the relative abundance of *Genus* *Bacteroides*.

Supplementary Figure 9. Network plots highlight the correlations between significant genera in Group N and Group P.

The correlation is measured by Spearman correlation coefficient. Lines between nodes represent correlations between the connected nodes, with linewidth indicating the correlation magnitude. Red line represents positive correlation while green line represents negative correlation. For clarity, only the correlation network between the significant genera that included the first 13 showing large relative abundance among Groups N and P is calculated. Only the correlations with statistical difference are included in the network.

Supplementary Figure 10. Different metabolic profiling between group N and group P shown by OPLS-DA.(A) Score scatter plot of OPLS-DA model for group N vs P in the POS model. (B) Permutation test of OPLS-DA model for group N vs P in the POS model. (C) Score scatter plot of OPLS-DA model for group N vs P in the NEG model. (D) Permutation test of OPLS-DA model for group N vs P in the NEG model.

Supplementary Figure 11. Correlation between microbiota and metabolic profiling.

Matrix of correlation coefficient (Corr) of differentiated metabolites between Group P and Group N is calculated by using the “spearman” algorithm. Heatmap indicates the correlation between intestinal flora profiling and metabolic profiling with red indicated a Corr of 1 while blue indicated a Corr equaled to -1. Data of the Corr with statistical difference were marked with “*” in the graph. The differentiated intestinal flora is shown in vertical coordinate while differentiated metabolites are shown in the horizontal coordinate. (A) Matrix of Corr under positive ion (POS) mode. (B) Matrix of Corr under negative ion (NEG) mode. Correlation network indicates the correlation between microbiota and metabolites. Red indicates a Corr of 1 while blue indicates a Corr equalled to -1. Differentiated metabolites are shown in green and differentiated intestinal flora are shown in red. (C) Correlation network under POS mode. (D) Correlation network under NEG mode.

Correlation network was constructed by using Cytoscape_v3.7.1 software (https://cytoscape.org/download.html).
