# Supplementary material for: Meta-omics characteristics of intestinal microbiota associated to HBeAg seroconversion induced by oral antiviral therapy
Source: Sci Rep. 2021 Feb 5;11:3253. doi: 10.1038/s41598-021-82939-1 (PMC7864979; doi:10.1038/s41598-021-82939-1)
Supplement: Supplementary file 13 — Supplementary Tables. [file 41598_2021_82939_MOESM13_ESM.docx]

## Meta-Omics Characteristics of Intestinal Microbiota Associated to HBeAg Seroconversion Induced by Oral Antiviral Therapy

## Running title: Microbiota and HBeAg Seroconversion

Yu-Li Zeng, MD^1†^, Lei Qin, PhD^2†^, Wen-Jun Wei, MD^1†^, Hong Cai, MD^3^, Xiao-Fang Yu, MD^3^, Wei Zhang, MD^4^, Xiao-Lu Wu, MD^5^, Xiao-Bin Liu, MD^5^, Wei-Ming Chen, MD^1^, Pan You, MD, PhD^6^, Mei-Zhu Hong, MD^7^, Yaming Liu, MD, PhD^1^, Ben-Chang Shia, PhD^8*^, Jian-Jun Niu, MD, PhD^6*^ and Jin-Shui Pan, MD, PhD^9*^

^1^Department of Gastroenterology, Zhongshan Hospital Xiamen University, Xiamen, Fujian, China

^2^School of Statistics, University of International Business and Economics, Chaoyang District, Beijing, China

^3^Department of Hepatology, Xiamen Hospital of Traditional Chinese Medicine, Xiamen, Fujian, China

^4^Department of Research Institute, the Fifth Hospital of Shijiazhuang, Shijiazhuang, Hebei, China

^5^Department of Infectious Diseases, the First Affiliated Hospital of Xiamen University, Xiamen, Fujian, China

^6^Center of Clinical Laboratory, Zhongshan Hospital Xiamen University, Xiamen, Fujian, China

^7^Department of Traditional Chinese Medicine, Zhongshan Hospital Xiamen University, Xiamen, Fujian, China

^8^School of Management & Big Data Research Centre, Taipei Medical University, Taipei, Xinyi, Taiwan

^9^Liver Research Center, the First Affiliated Hospital of Fujian Medical University, Fuzhou, Fujian, China;

^†^These authors contributed equally.

^*^**Correspondence:**

Jin-Shui Pan, MD, PhD, E-mail: [j.s.pan76@gmail.com](mailto:j.s.pan76@gmail.com);

Jian-Jun Niu, MD, PhD, E-mail: [niujianjun62@163.com](mailto:niujianjun62@163.com);

Ben-Chang Shia, PhD, E-mail: [stat1001@tmu.edu.tw](mailto:stat1001@tmu.edu.tw)

Supplementary Table 1. The detailed eligibility and exclusion criteria for the study.

| ***Eligible criteria*** |
| --- |
| Eligible subjects were 18-50 years old |
| HBeAg was positive while anti-HBeAg was negative before oral antiviral therapy |
| Ongoing ETV- or TDF-based antiviral therapy |
| 18.5 ≤ body mass index < 27.0 |
| HBeAg seroconversion occurred at 12-156 weeks after ETV- or TDF-based therapy or HBeAg remained positive after more than 156 weeks of ETV- or TDF-based therapy |
| International normalized ratio (INR) ≤ 1.5 |
| ***Exclusion criteria*** |
| Companied by alcoholism; genetic or metabolic liver diseases, such as Wilson’s disease and hemachromatosis |
| Co-infection with HCV, HDV, HIV, EBV, or CMV |
| Concurrent malignant tumors; autoimmune liver diseases; liver cirrhosis; moderate or severe nonalcoholic fatty liver disease or non-alcoholic steatohepatitis confirmed by ultrasound, computed tomography, or FibroScan |
| Pregnant or lactating women |
| Concurrent conditions such as diabetes mellitus, hypertension with blood pressure ≥ 160/100 mmHg, heart failure, and renal impairment |
| History of acute gastroenteritis in the last 8 weeks before enrollment |
| Patients who had a history of other antiviral treatment such as interferon or pegylated interferon, lamivudine, adefovir, or telbivudine were excluded |
| Patients who had previous usage of immunomodulatory, antibiotics, and probiotics in the last 8 weeks were also excluded |

BMI, body mass index; ALT, alanine aminotransferase; AST, aspartate aminotransferase; ETV, entecavir; TDF, tenofovir disoproxil fumarate.

Supplementary Table 2. Clinical and Demographic Characteristics for the Enrolled Patients with CHB with or without HBeAg Seroconversion

| ***Median (range)*** | ***Group H*** | ***Group N*** | ***Group P*** | ***P value*** |
| --- | --- | --- | --- | --- |
| Age, year | 32.0 (18-45) | 34.0 (20-49) | 36.0 (22-50) | 0.0719^*^ |
| Sex, male/female | 10/6 | 24/13 | 33/8 | 0.2183^†^ |
| BMI | 22.45 (18.23-26.56) | 21.85 (18.57-26.87) | 22.10 (18.36-26.93) | 0.7466^*^ |
| HBV DNA (log_10_) |  | 6.81 (4.91-9.00) | 7.68 (4.42-9.70) | 0.0314^§^ |
| ALT, IU/L |  | 197.1 (16.0-1166.0) | 140.9 (19.0-936.0) | 0.0769^§^ |
| AST, IU/L |  | 91.2 (11.0-839.0) | 81.3 (6.0-768.0) | 0.2083^§^ |
| Treatment, ETV/TDF |  | 20/17 | 25/16 | 0.6474^†^ |

Group N, who achieved HBeAg seroconversion at 12-156 weeks after ETV- or TDF-based therapy;

Group P, who remained HBeAg positive after more than 156 weeks of ETV- or TDF-based therapy;

BMI, body mass index; ALT, alanine aminotransferase; AST, aspartate aminotransferase; ETV, entecavir; TDF, tenofovir disoproxil fumarate.

^*^Comparison in three groups using nonparametric test, Kruskal-Wallis test.

^†^Comparison using chi-square test or Fisher's exact test.

^§^Comparison using nonparametric test, Mann Whitney test.

Supplementary Table 3. The abundance, taxonomy, and importance of significant genus.

| ***Genus*** | ***Abundance in group N*** | ***Abundance in group P*** | ***P Value*** |  | ***Importance of Genus*** | ***Importance for N Vs P*** |
| --- | --- | --- | --- | --- | --- | --- |
| *Acidaminococcus* | 0.001231 | 0.000036 | 0.0141 |  | 3.169583 | 0.001195 |
| *Bacteroides* | 0.305186 | 0.413659 | 0.0166 |  | 4.364811 | -0.108473 |
| *Christensenella* | 0.000008 | 0.000019 | 0.0373 |  | 0.842384 | -0.000010 |
| *Clostridium* | 0.003728 | 0.001362 | 0.1416 |  | 3.652585 | 0.002366 |
| *Dehalobacterium* | 0.000038 | 0.000015 | 0.0438 |  | 1.663051 | 0.000023 |
| *Eubacterium* | 0.000116 | 0.000236 | 0.3380 |  | 2.634557 | -0.000120 |
| *Mitsuokella* | 0.000371 | 0.000004 | 0.0680 |  | 0.879391 | 0.000367 |
| *Oscillospira* | 0.009609 | 0.013128 | 0.0872 |  | 5.103378 | -0.003519 |
| *Sutterella* | 0.014269 | 0.027627 | 0.0101 |  | 4.651063 | -0.013357 |
| *un_f_Clostridiaceae* | 0.004504 | 0.001502 | 0.4244 |  | 4.582411 | 0.003002 |
| *un_f_Erysipelotrichaceae* | 0.000813 | 0.000869 | 0.8554 |  | 3.294338 | -0.000056 |
| *un_f_Prevotellaceae* | 0.000006 | 0.001779 | 0.1917 |  | 0.762612 | -0.001773 |
| *un_o_Streptophyta* | 0.000254 | 0.000136 | 0.2679 |  | 2.422413 | 0.000118 |

Group N, who achieved HBeAg seroconversion at 12-156 weeks after ETV- or TDF-based therapy;

Group P, who remained HBeAg positive after more than 156 weeks of ETV- or TDF-based therapy.

Supplementary Table 4. Loadings of microbial signatures and clinical parameters along the first co-inertia component (PC1) and the second co-inertia component (PC2)

| ***Parameters*** | ***PC1*** | ***PC2*** |
| --- | --- | --- |
| *Acidaminococcus* | 0.0525 | 0.3365 |
| *Bacteroides* | -0.1579 | -0.3559 |
| *Christensenella* | -0.2116 | -0.1567 |
| *Clostridium* | -0.1582 | 0.3037 |
| *Dehalobacterium* | -0.0954 | 0.3310 |
| *Eubacterium* | 0.1493 | -0.2958 |
| *Mitsuokella* | 0.0605 | 0.1550 |
| *Oscillospira* | -0.1072 | -0.1897 |
| *Sutterella* | -0.2240 | -0.0263 |
| *un_f_Clostridiaceae* | -0.1108 | -0.0872 |
| *un_f_Erysipelotrichaceae* | -0.0171 | -0.3733 |
| *un_f_Prevotellaceae* | -0.2662 | 0.0564 |
| *un_o_Streptophyta* | 0.0835 | -0.0645 |
| Age, year | -0.1655 | -0.1376 |
| BMI | -0.1511 | 0.0919 |
| HBV DNA | -0.1855 | -0.2411 |
| AST, IU/L | 0.5245 | -0.1597 |
| ALT, IU/L | 0.5092 | -0.1576 |
| Time, month^*^ | -0.3105 | -0.3089 |

^*^Time refers to the time when a specified individual acquires HBeAg seroconversion after initiation of oral antiviral therapy;

PC, principal component; BMI, body mass index; ALT, alanine aminotransferase; AST, aspartate aminotransferase.

Supplementary Table 5. Significant correlations (*P* < 0.05) between microbial signatures and clinical parameters

|  | ***PC1*** | ***PC2*** | ***un_f_Prevotellaceae*** | ***Sutterella*** | ***Bacteroides*** | ***Age, year*** | ***AST, IU/L*** |
| --- | --- | --- | --- | --- | --- | --- | --- |
| PC1 |  |  |  |  |  |  |  |
| PC2 | - |  |  |  |  |  |  |
| *un_f_Prevotellaceae* | -0.4184 | 0.2213 |  |  |  |  |  |
| *Sutterella* | -0.3530 | - | - |  |  |  |  |
| *Bacteroides* | - | -0.5647 | - | 0.4004 |  |  |  |
| *un_f_Erysipelotrichaceae* | - | -0.5490 | - | - | - |  |  |
| Age, year | -0.2598 | - | - | - | 0.2555 |  |  |
| BMI | -0.2563 | - | - | - | - | 0.2269 |  |
| HBV DNA | -0.3070 | -0.3548 | - | - | - | - |  |
| AST, IU/L | 0.8354 | -0.2523 | -0.3570 | - | - | - |  |
| ALT, IU/L | 0.8060 | -0.2542 | -0.3077 | - | - | - | 0.8798 |
| Time, month^*^ | -0.5108 | -0.4911 | - | - | 0.2318 | 0.2664 | -0.2333 |

^*^Time refers to the time when a specified individual acquires HBeAg seroconversion after initiation of oral antiviral therapy;

PC, principal component; BMI, body mass index; ALT, alanine aminotransferase; AST, aspartate aminotransferase.
